# A MYB-related transcription factor from peanut, AhMYB30, improves freezing and salt stress tolerance in transgenic *Arabidopsis* through both DREB/CBF and ABA-signaling pathways

**DOI:** 10.3389/fpls.2023.1136626

**Published:** 2023-02-28

**Authors:** Na Chen, Lijuan Pan, Zhen Yang, Maowen Su, Jing Xu, Xiao Jiang, Xiangzhen Yin, Tong Wang, Feifei Wan, Xiaoyuan Chi

**Affiliations:** ^1^ Key Laboratory of Peanut Biology, Genetic & Breeding, Ministry of Agriculture and Rural Affairs, Shandong Peanut Research Institute, Qingdao, China; ^2^ Department of Animal and Plant Quarantine, Qingdao Customs, Qingdao, China; ^3^ Division for Guidance of Cooperative Economy, Binzhou Agricultural Technology Extension Center, Binzhou, China

**Keywords:** peanut (*Arachis hypogaea* L.), MYB-related transcription factor, cold and salt stress, DREB/CBF signaling pathway, ABA-signaling pathway

## Abstract

Abiotic stresses such as salinity and low temperature have serious impact on peanut growth and yield. The present work investigated the function of a MYB-related transcription factor gene *AhMYB30* obtained from peanut under salt and low temperature stresses by transgenic methods. The results indicated that the overexpression of *AhMYB30* in *Arabidopsis* could enhance the resistance of transgenic plants to freezing and salt stresses. The expression of stress-response genes *RD29A* (*Response-to-Dehydration 29A*), *COR15A* (*Cold-Regulated 15A*), *KIN1* (*Kinesin 1*) and *ABI2* (*Abscisic acid Insensitive 2*) increased in transgenic plants compared with in wild-type. Subcellular localization and transcriptional autoactivation validation demonstrated that AhMYB30 has essential features of transcription factors. Therefore, AhMYB30 may increase salt and freezing stress tolerance as the transcription factor (TF) in *Arabidopsis* through both DREB/CBF and ABA-signaling pathways. Our results lay the theoretical foundation for exploring stress resistance mechanisms of peanut and offering novel genetic resources for molecular breeding.

## Introduction

Abiotic stresses, like salt, cold and drought, have adverse impacts on plant growth and yield. For adapting to such stresses, plants evolve complicated signaling cascades to regulate the expression of stress-related genes involved in the direct or indirect improvement of stress resistance (Huang et al., 2012; Ritonga et al., 2021). In this process, the transcription factors (TFs) play a crucial role (Khan et al., 2018). As demonstrated in previous studies, some TF genes, including ERF/DREB, MYB and bZIP, have critical effects on regulating abiotic stresses in plants (Baillo et al., 2019). For example, the role of CBFs in enhancing cold/freezing tolerance in plants has been well established in many species (Artlip et al., 2014; Ritonga et al., 2021). Many MYB genes isolated from *Arabidopsis*, rice or other plants were proved to be involved in plant cold, dehydration or salt stress (Wang et al., 2021).

MYB TFs are found to have important effects on regulating plant growth, responses to biotic/abiotic stress and metabolism (Cao et al., 2020; Wang et al., 2021). The previous researches regarding MYB genes mostly emphasize on R2R3-MYB gene family, and it is demonstrated to have critical effects on numerous plant-specific processes like abiotic stress responses (Wang et al., 2021). Overexpression of the R2R3-MYB genes of *Arabidopsis*, including *AtMYB41*, *AtMYB44* and *AtMYB61*, is capable of increasing resistance to abiotic stress in transgenic plants *via* different regulation mechanisms. AtMYB41 may respond to drought, salt and cold stresses by participating in the regulation of epidermal synthesis and cell proliferation (Cominelli et al., 2008; Lippold et al., 2009; Hoang et al., 2012). AtMYB44 and AtMYB61 can prompt stomatal closure in transgenic plants to increase resistance to drought and salt stresses (Liang et al., 2005; Jung et al., 2008). *OsMYB2* is responsible for encoding one R2R3-MYB TF, which elevates proline and soluble sugar levels to enhance high salt, drought, and low temperature stress resistance of transgenic rice (Yang et al., 2012).The wheat R2R3-MYB gene*TaMYB19-B*, which could improve salt, drought and freezing stress resistance by up-regulating some stress-responsive genes when exposed to exogenous ABA and abiotic stress treatments (Zhang et al., 2014). ZmMYB31, a R2R3-MYB transcription factor in maize, positively regulates the expression of *CBF* genes and enhances resistance to chilling and oxidative stress (Li et al., 2019). Soybean R2R3 MYB gene *GmMYB81* confers plant tolerance to salt and drought stress during seed germination through interacting with the abiotic stress regulator *GmSGF14l* (Bian et al., 2020). Many R2R3 MYB genes isolated from different plants are all proved to participate salt, drought or low temperature stress regulation in transgenic plant through different function mechanisms (Huang et al., 2018; Shen et al., 2018; Dong et al., 2021; Zhou et al., 2022). Some R2R3 MYB proteins negatively regulate plant abiotic stresses. For example, *AtMYB73* of *Arabidopsis* is induced by salt stress at the transcriptional level, while its deletion mutant show an enhanced salt tolerance phenotype than wild-type plant, indicating that this protein is a negative regulator in the regulation of *Arabidopsis* salt stress resistance (Kim et al., 2013).*TaMpc1-D4*, the wheat R2R3 MYB gene, shows adverse regulation on transgenic wheat and *Arabidopsis* in terms of their drought resistance through modulating antioxidant- and stress-related gene levels and enzyme system capacity (Li et al., 2020).

Previous studies of the MYB-related type proteins have concentrated on their regulation of circadian rhythm and cell morphogenesis (Kirik et al., 2004; Nguyen and Lee, 2016). However, in recent years, more and more MYB-related type genes are demonstrated with important functions in regulating abiotic stress responses in plants. OsMYB48-1 is a new MYB-related TF, and it can regulate stress-induced Abscisic acid (ABA) generation to positively affect salt and drought stress resistance (Xiong et al., 2014). Two MYB-related type genes, *LcMYB1* and *LcMYB2*, in sheepgrass are demonstrated to increase the salt and drought resistance of transgenic *Arabidopsis*, separately (Cheng et al., 2013; Zhao et al., 2019). *LcMYB1* increases salt stress tolerance of transgenic *Arabidopsis* through increasing the amounts of proline and soluble sugars under salt stress conditions (Cheng et al., 2013). In transgenic *Arabidopsis*, LcMYB2 improves plant drought stress through the direct combination with respective promoters to activate osmotic stress-related gene levels of *AtDREB2A*, *AtLEA14* and *AtP5CS1* (Zhao et al., 2019). In addition, LlMYB3 in tiger lily and OsMYB-R1 in rice are both proved to be involved in plant abiotic stress (Yong et al., 2019; Tiwari et al., 2020). At present, function studies about MYB-related proteins in plant abiotic stress are scarce. With the deepening of research, it is believed that more and more MYB-related proteins will be confirmed to be involved in plant abiotic stress regulation.

As a result, the MYB transcription factor family plays important effects on regulating abiotic stress resistance in plants, but the functions and mechanisms of the different genes are not identical. Peanut (*Arachis hypogaea* L.), which is the main edible oil source, is an important oil seed crop (Pruthvi et al., 2014). Cold stress seriously influences its germination, development, bloom and yields (Wang et al., 2003; Sorensen et al., 2009; Upadhyaya et al., 2009; Liu et al., 2017; Chang et al., 2019). Salinity decreases peanut germination and dry matter production (Singh et al., 2010; Meena et al., 2016), damaging the photosynthetic apparatus (Qin et al., 2011), and inducing K and Ca deficiencies (Leidi et al., 1992) and important yield losses (Singh et al., 2010). Nevertheless, there have been limited studies conducted on MYB transcription factor genes, especially the MYB-related type, in peanut. MYB gene family was comprehensively analyzed within peanut, as a result, 8 MYB genes were found to participate in regulating plant abiotic stress resistance. Among them, cold and salt stresses induced MYB-related gene *AhMYB30* expression (Chen et al., 2014). Based on the preliminary research, this work analyzed *AhMYB30*’s role in plant freezing and salt stress regulation, and the possible mechanism of action was discussed.

## Materials and methods

### Plant materials

The peanut variety used for gene cloning was cultivar Huayu 33. The wild-type *Arabidopsis thaliana* adopted for transformation was Columbia ecotype.

### RNA isolation and cDNA synthesis

Total RNA was isolated from leaves of peanut Huayu 33 by TRIzol® Reagent (Invitrogen, Carlsbad, CA, USA). The cDNA synthesis was performed using EasyScript^®^ First-Strand cDNA Synthesis SuperMix (TransGen, Beijing, China) in a 20 μL reaction solution containing 2 μg total RNA. Reverse transcription was conducted for 30 min at 42°C, and the mixture was stored at -20°C.

### Cloning of *AhMYB30* gene

PCR amplification was conducted to amplify full-length coding sequence (CDS) of *AhMYB30* (Gene ID: KF208684), using gene-specific primers: 5’-ATGAAGTGGGAAGTAGAAGTA-3’ and 5’-CTATAATATAAGTGAAGAAAA-3’. PCR was performed in the 50 μL reaction system (Vazyme, Nanjing, China), containing 22 μL double-distilled water, 25 μL of 2 × Phanta Max Master Mix, 1 μL of each primer (10 μM) and 1 μLcDNA sample. Later, 1% agarose gel electrophoresis (AGE) was conducted to separate PCR products, while Gel Extraction Kit (Axygen, California, USA) was employed for product purification in line with specific protocols. The purified fragments were stored at -20°C.

### Subcellular localization

We ligated AhMYB30 CDS between the CaMV 35S promoter and eGFP CDS in pCAMBIA1300-eGFP vector, and thus the candidate protein was fused with GFP at its C-terminus. DAPI (4’,6-Diamidino-2-Phenylindole, Dihydrochloride), as a blue fluorescent dye, was employed to label cell nucleus. According to previous reports (Sparkes et al., 2006), tobacco leaf transient expression system was performed to detect subcellular localization of candidate protein. On the third day after infiltration, FluoView FV1000 confocal microscope (Olympus, Tokyo, Japan) was employed for fluorescence observation at the emission and excitation wavelengths of 510 and 488 nm separately for GFP, whereas 460 and 360 nm for DAPI.

### Validation of AhMYB30 transcriptional activity

AhMYB30 transcriptional activity was analyzed by yeast strain AH109 by adopting *LacZ* and *HIS3* reporter genes. This work amplified full-length CDS of AhMYB30 using RT-PCR with primers of 5’-GAATTCATGAAGTGGGAAGTAGAAGTA-3’ (*EcoR*I site underlined) and 5’-CTGCAGCATTTGGAAAGCAATGTTTTG-3’ (*Pst*I site underlined).

The validation method for transcriptional activity was described previously (Ma et al., 2009). *EcoR*I/*Pst*I was added to digest PCR products, followed by insertion in pGBKT7 to construct the AhMYB30–pGBKT7 expression vector. By adopting lithium acetate-mediated approach, the pGBKT7 (negative control) and AhMYB30–pGBKT7 plasmids were independently transfected in yeast AH109 cells (Gietz et al., 1992), while SD/–Trp auxotrophic medium was utilized to screen transformants. In addition, SD/–Trp/–His/–Ade auxotrophic medium was utilized to streak positive transformants, followed by growth observation at 3 days later. The galactosidase assay was performed according to a previously described method (Ma et al., 2009).

### Vector construction and *Arabidopsis* transformation

At first, the vector pCAMBIA1300 was linearized using *BamH*I and *Xba*I (NEB, Ipswich, England). Then, RT-PCR was conducted to amplify *AhMYB30* gene by using forward primer 5′-AACATGTCGACACGTGGATCCATGAAGTGGGAAGTAGAAGTA-3′ (*BamH*I site underlined) and the reverse primer 5′-GCGCTCAGTTGGAATTCTAGACTATAATATAAGTGAAGAAAA-3′ (*Xba*I site underlined). The 5 ‘end of forward or reverse primer was homologous with either end of the linearized vector pCAMBIA1300, respectively. After electrophoretic recovery of the PCR products, Infusion reactions were performed using ClonExpress Ultra One Step Cloning Kit (Vazyme, Nanjing, China) for constructing an overexpression vector.

The overexpression plasmids were transformed in Agrobacterium tumefaciens C58, and subsequently in *Arabidopsis* by a floral dip approach (Clough and Bent, 1998). The seeds of T_1_ generation were sterilized using 10% NaClO and 0.2% Triton-X100 for 5 min before washing with distilled water 5 times. Later, this work uniformly distributed those sterilized seeds onto the half strength of Murashige and Skoog (1/2 MS) medium that contained 30 mg L^–1^ hygromycin. The plants screened positive for resistance were later placed in the soil pots for growth under 22°C and 16h/8h light/dark cycle conditions.

### Freezing and salt stress treatments of transgenic *Arabidopsis*

The freezing and salt experiments for transgenic plants were performed according to a previous report (Zhou et al., 2009) with slight modifications in the growth time (Freezing: 20 d compared to 4 weeks; salt: 3 d compared to 2 d) and treatment time (Freezing: –8°C for 1 h, 3 h, 6 h compared to –6°C for 6 h; salt: 5 d compared to 5~6 d).

This work later scattered *AhMYB30* transgenic and WT *Arabidopsis* line seeds into the soil containing 1:2 mixture of perlite to vermiculite, for growth under 22°C and 16-h/8-h light/dark cycle conditions. After ~20 d, the plants were put into a biochemical incubator (Yiheng, Shanghai, China) with light, and the temperature was decreased to –8°C for 1 h, 3 h, 6 h. Subsequently, the freezing treated materials were placed under 22°C and 16-h/8-h light/dark cycle conditions to recover and the phenotype were observed.

The seeds of three *AhMYB30* overexpression transgenic lines and wild-type *Arabidopsis* were placed at 4°C for 3 d to break dormancy, and later spread on ½ MS medium. *Arabidopsis* seedlings of all genotypes were transferred to ½ MS medium containing 0, 100, 150 and 200 mM NaCl respectively at 3 d after germination. *Arabidopsis* root elongation was measured and the leaf color variation was observed after 5 d of cultivation in an illuminated incubator (Yiheng, Shanghai, China). All of the treatments were performed at least three replicates.

### Quantitative fluorescent PCR

*AhMYB30* together with stress-related genes was analyzed within *Arabidopsis* using quantitative fluorescent PCR. The PCR reaction was carried out in line with Chen et al.’s approach (Chen et al., 2012). The cDNA samples were diluted to 8 ng μL^-1^. Triplicate quantitative assays were performed with 2 μL of each cDNA dilution with SYBR Premix Ex Taq polymerase (Takara, Tokyo, Japan) by using a LightCycler 2.0 instrument system (Roche, Germany) according to the manufacturer’s protocol. The reactions were subjected to an initial denaturation step of 95°C for 10 s, followed by 40 cycles of 95°C for 5 s, 60°C for 30 s and 72°C for 10 s. A melting curve analysis was performed at the end of the PCR, increasing the temperature stepwise by 0.5°C every 10 s. Raw data were later transformed in relative quantities by the delta-Cq approach according to previous study (Chi et al., 2012). The reference gene was *Arabidopsis Actin2*. The PCR primers of reference and all genes are listed in **Table 1**.

**Table 1:** Primers for fluorescent quantitative PCR.

Gene name	Forward primers (5’-3’)	Reverse primers (5’-3’)
*Actin*	GGTAACATTGTGCTCAGTGGTGG	AACGACCTTAATCTTCATGCTGC
*AhMYB30*	ATGGGGACTTATGATCAA	TCATCCTTCAACGTTTGC
*ICE1*	TCCTAAAGGCCAGCAAGCT	CTCTTGTCCTTCTTGGCATTG
*CBF1*	CTTCGCTGACTCGGCTTGG	ACGCACCTTCACTCTGTTCC
*CBF2*	AACCAGCGGGAAGGAAGAAGT	TTTCCTTGGCAC AGGTTGATT
*DREB1A/CBF3*	GATCAGCCTGTCTCAATTTC	CTTCTGCCATATTAGCCAAC
*COR47*	TATCATGCCAAGACCACTGAA	CAACGAAAGCCACAATAACAA
*COR15A*	CTCAGTTCGTCGTCGTTTC	CATCTGCTAATGCCTCTTT
*P5CS*	GGTGGACCAAGGGCAAGTAAGA TA	TCGGAAACCATCTGAGAATCTTGT
*RD29A*	GGCGTAACAGGTAAACCTAGAG	TCCGATGTAAACGTCGTCC
*KIN1*	ACCAACAAGAATGCCTTCCA	CCGCATCCGATACACTCTTT
*ABI2*	AGGCTATTGCAACGGTGTGG	TCTGGTCGTCTACCACAAATCG
*Actin2*	GGAAGGATCTGTACGGTAAC	TGTGAACGATTCCTGGACCT

## Results

### AhMYB30 has the basic characteristics of a transcription factor

**Figure 1:**
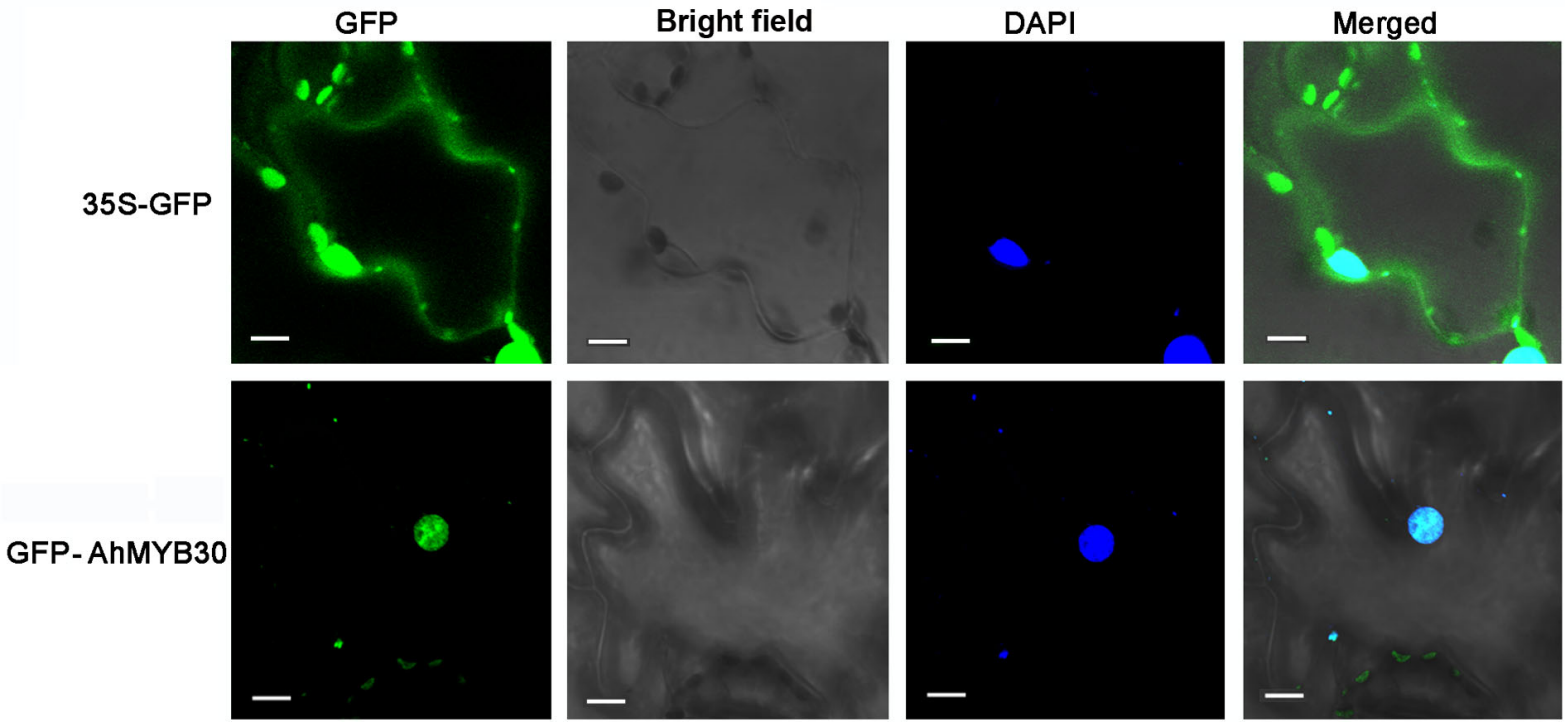
Subcellular localization of the AhMYB30-GFP fusion protein. Fluorescence microscopic images of tobacco epidermal cells transformed with 35S::AhMYB30-GFP and 35S::GFP. Scale bars for 35S::GFP and 35S::AhMYB30-GFP are 10 μm.

GFP control andAhMYB30-GFP fusion construct in pCAMBIA1300-eGFP vector driven by CaMV35S promoter were transiently expressed in tobacco epidermal cells. Then the subcellular localization of AhMYB30 were observed under a laser scanning confocal microscope. As a result, the fluorescence signals from GFP alone were widely distributed throughout the cells. Whereas, the AhMYB30-GFP fusion protein fluorescence signal was mainly detected in the nucleus (**Figure 1**).

**Figure 2:**
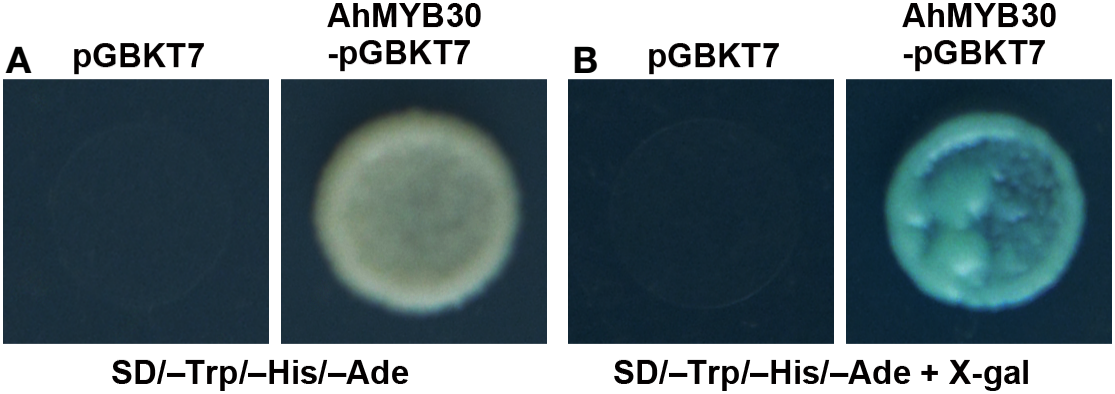
Transcription activity analysis of full-length AhMYB30 protein. **(A)**, Growth of transformants with pGBKT7 (left) and AhMYB30-pGBKT7 (right) on auxotroph medium (SD/–Trp/–His/–Ade); **(B)**, X-gal activation detection of transformed yeast on the SD/–Trp/–His/–Ade plates.

A yeast system was adopted for analysis of AhMYB30’s transcriptional activity. The full-length cDNA of *AhMYB30* was fused with GAL4 DNA-binding domain within pGBKT7, and the fusion plasmid AhMYB30–pGBKT7 was transformed into yeast strain AH109. The growth condition on SD/–Trp/–His/–Ade triple-auxotrophic medium and the results of the galactosidase activity experiment are displayed in **Figure 2**. The yeast transformed with negative control (pGBKT7 empty vector) showed no growth on the triple-auxotrophic medium. However, the yeast transformed with the AhMYB30–pGBKT7 vector could grow on the triple-auxotrophic medium and turned blue in the galactosidase activity assay.

The above results indicated that AhMYB30 is a nuclear protein and has transcriptional activity in yeast.

### Overexpression of *AhMYB30* in *Arabidopsis* could increase resistance of transgenic plants to freezing and salt stresses

**Figure 3:**
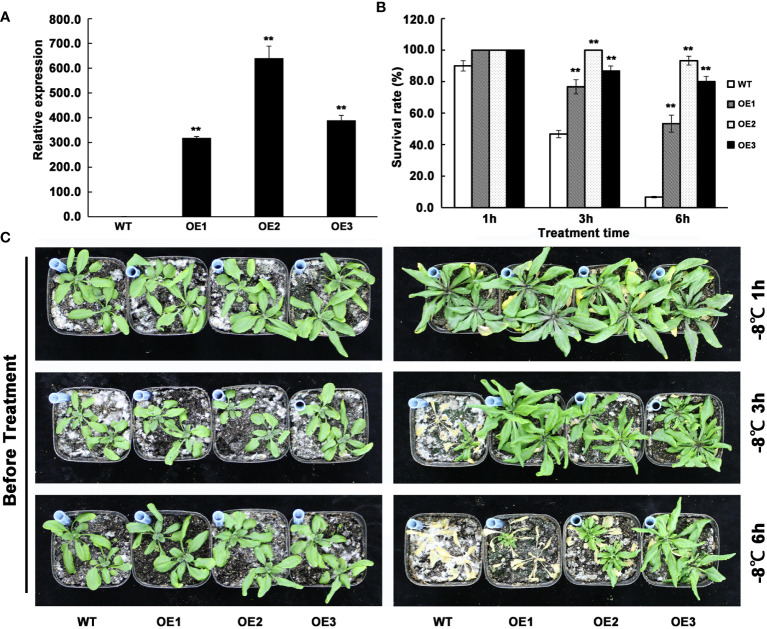
Detection of *AhMYB30* transgenic *Arabidopsis* plants and phenotype observation of the transgenic plants under freezing stress. **(A)**, quantitative fluorescent PCR analysis of *AhMYB30* expression in wild type controls (WT) and three independent transgenic lines of *Arabidopsis* (OE1, OE2, and OE3). *Arabidopsis Actin2* transcript was amplified as a control; **(B)**, Quantitative analysis of the plant survival rate 14 d after the freezing treatment as shown in **(C)**. Error bars indicate standard deviation (SD), which was calculated using results of three replicated experiments. Asterisks indicate a significant difference (^**^ p < 0.01) compared with the corresponding controls; **(C)**, Phenotype of wild-type (WT) and *AhMYB30*-overexpressed plants after freezing stress. Twenty-day-old WT and transgenic plants were freezing stressed at -8°C for 1, 3, and 6 h and then transferred back to the normal condition for recovery.

To investigate the function of *AhMYB30* in plant abiotic stresses, the cDNA was over-expressed under the driven of the CaMV35S promoter in *Arabidopsis*. The transgenic lines were screened using hygromycin and validated using quantitative fluorescent PCR (**Figure 3A**). Three lines with significantly increased *AhMYB30* expression levels were selected (**Figure 3A**). For those 3 *AhMYB30*-overexpressed plants, T_3_ progeny was generated and no obvious differences in phenotype or development occurred between transgenic and WT *Arabidopsis* lines in the normal growth environment (**Figure 3C** left).

Following a freezing-stress treatment, transgenic and WT *Arabidopsis* plants grow in the normal environment, with their phenotypes being observed after 7 d. When treated 1 h by -8°C, the survival rate of WT was slightly lower than the transgenic plants during the recovery process. However, when treated 3 h by -8°C,the wild-type plants grew slower than transgenic plants and the survival rate was less than 50%, while the survival rate of transgenic lines were all more than 70%. When treated 3 h by -8°C, the survival rate of wild-type plants was significantly reduced to below 10%, while the survival rate of all three transgenic plants was still above 50% (**Figures 3B, C**). The above phenotypes suggested that *AhMYB30* overexpression within plants promoted freezing stress tolerance in transgenic plants.

**Figure 4:**
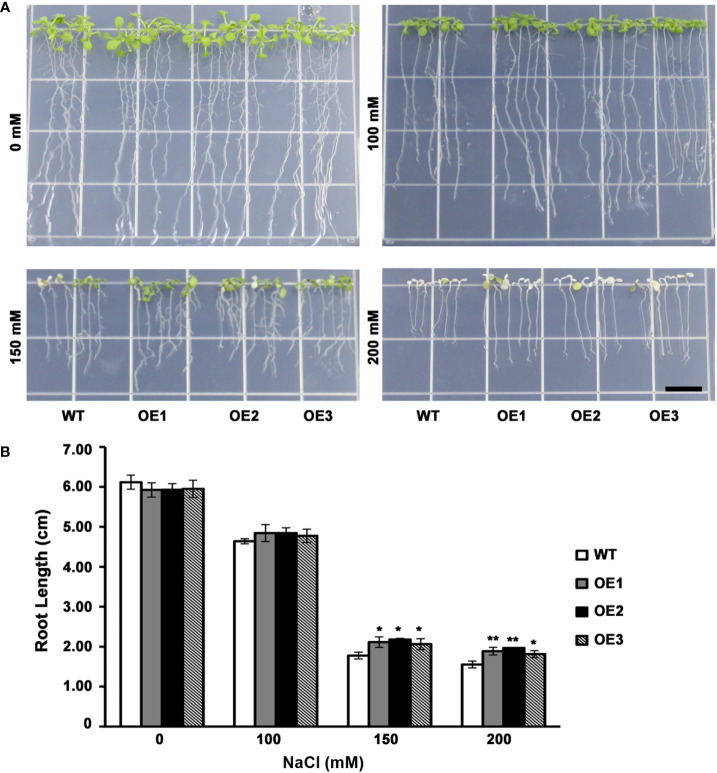
Effect of *AhMYB30* expression on salt tolerance in transgenic *Arabidopsis* plants. **(A)**, Phenotype of WT and transgenic plants after salt stress. Wild-type and transgenic plants were germinated on ½MS agar plates for 3 d, then transferred to a new ½MS agar plate supplemented with different concentrations of NaCl for 5d; **(B)**, Root length of transgenic and WT seedlings treated with NaCl. All assays were repeated at least three times. Error bars represent SD of results of three replicated experiments. Scale bar for root length is 1 cm. Asterisks indicate a significant difference from the wild type (WT): *, 0.01<P<0.05 (*t*-test); **, P<0.01 (*t*-test).

The leaf color of WT as well as 3 T_3_ transgenic lines had no significant difference when grown on medium without NaCl or 100 mM NaCl. However, when grown on medium containing NaCl (150 mM), certain WT lines had yellow leaves, whereas transgenic plants still had green leaves (**Figure 4A**). At the same time, transgenic plants had increased root length compared with WT lines (**Figure 4B**). On the medium containing NaCl (200 mM), WT plant had totally yellow leaves, while the leaves of a few transgenic plants remained green (**Figure 4A**). And transgenic plants had evidently increased roots compared with WT lines (**Figure 4B**). These results indicated a lower level inhibition of root elongation in *AhMYB30* transgenic *Arabidopsis* than in wild-type under salt stress. Additionally, the ability to maintain green leaves was also improved, and thus, peanut *AhMYB30* overexpression within transgenic *Arabidopsis* enhanced salt stress tolerance.

### AhMYB30 activates the expression of cold-responsive genes

**Figure 5:**
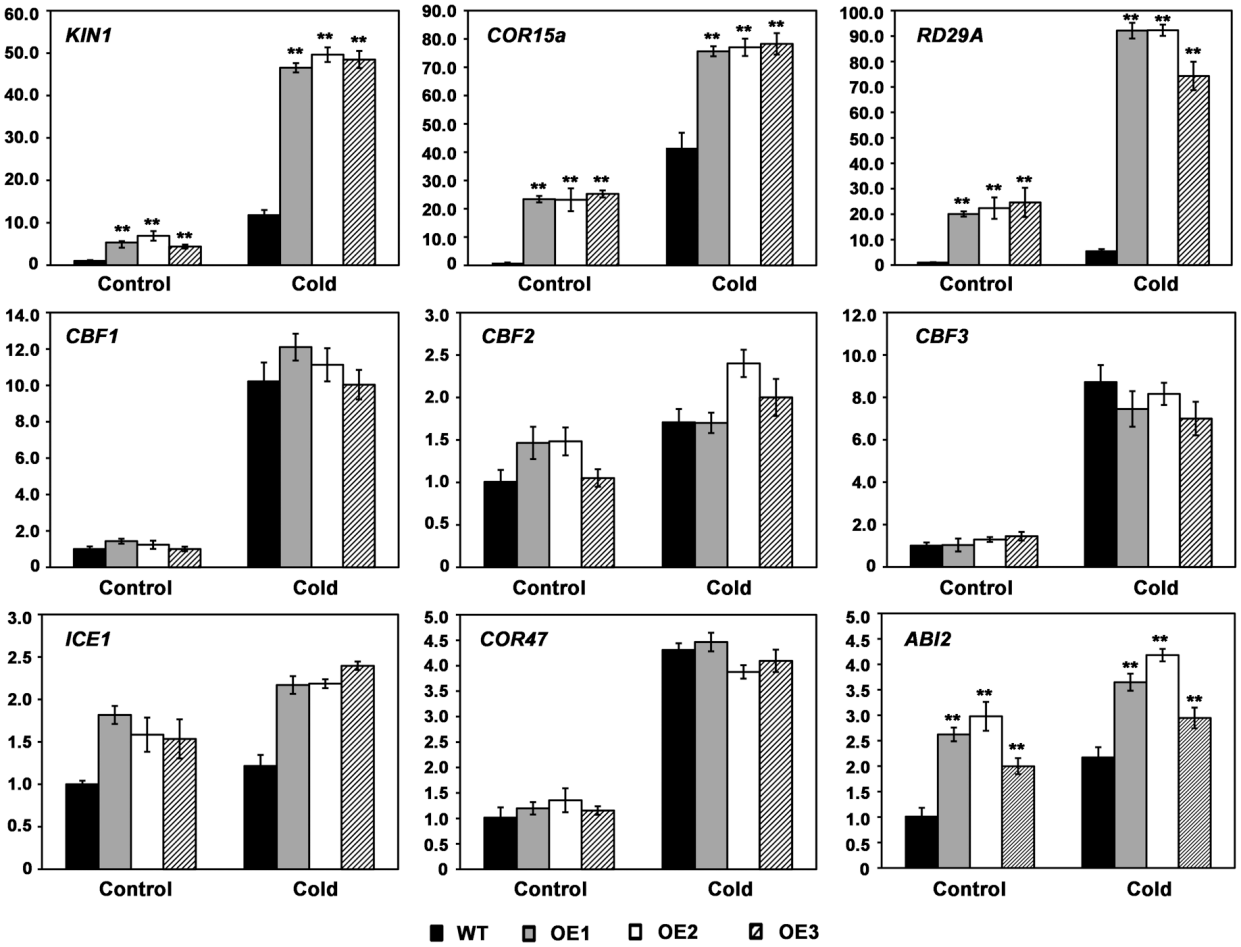
Expression patterns of stress responsive marker genes in wild-type and transgenic *Arabidopsis* using real-time PCR. Total RNA was extracted from 14-d-old plants grown under normal or cold treatment for 6 h. *Arabidopsis Actin2* was used as an internal control. Data represent means from three biological and three technical replicates, each containing three seedlings of each line. Error bars indicate SD. Asterisks indicate a significant difference from the wild type (WT): **, P<0.01 (*t*-test).

To elucidate the molecular mechanism of AhMYB30 in the cold response, cold-responsive gene levels related to regulatory pathways were detected with quantitative fluorescent PCR. Cold stress induced the expression of those measured marker genes, such as *CBF1*, *CBF2*, *CBF3*, *ICE1* (*Inducer of CBF expression 1*) and *COR47* (*Cold-Responsive 47*), however, there was no obvious difference between transgenic and WT lines upon cold-stress and control conditions (**Figure 5**). However, *AhMYB30*-overexpressed transgenic lines had markedly elevated *KIN1*, *COR15a*, *RD29A* and *ABI2* levels compared with WT lines under normal conditions (22°C). After 6h cold stress, their levels markedly increased within transgenic and WT lines, with markedly increased levels being detected in transgenic lines (**Figure 5**).

## Discussion

Gene transcripts that can encode some TF families, like AP2/ERF, NAC, and bHLH, can be induced by abiotic stress stimuli (Sun et al., 2018; Yoon et al., 2020; Ritonga et al., 2021). Such TFs are related to different pathways for conferring the plant resistance to stresses (Yoon et al., 2020). Typically, MYB family of proteins contains many members, with different functions, and they are mainly distributed in eukaryotes. These proteins can be detected in plants, which are related to ABA response as well as interaction with additional TFs (Ambawat et al., 2013). Members of MYB family have been increasingly suggested to have important effects on abiotic stress responses. Nonetheless, for many MYB genes, their enhanced stress tolerance is mostly obtained based on model species like rice and *Arabidopsis* (Dubos et al., 2010; Ambawat et al., 2013). Our previous study indicated that the expression of *AhMYB30* cloned from peanut (*Arachis hypogaea* L.) was induced by salt and cold stress (Chen et al., 2014). In this study, the function of *AhMYB30* was investigated under freezing and salt stresses.

Sequence analysis indicated that AhMYB30 belong to the MYB-related (one single SANT domain) subfamily (Chen et al., 2014). Compared to R2R3-MYB genes, few studies of the MYB-related genes in abiotic stress response have been reported in plants (Zhang et al., 2014). So far, no more than 10 MYB-related members including RSM1, LlMYB3, LcMYB2 and so on, are reported to be related to abiotic stresses of plants (Yang et al., 2018; Yong et al., 2019; Zhao et al., 2019). This work constructed the *Arabidopsis* transgenic plants with *AhMYB30* overexpression controlled by constitutive CaMV35S promoter. The stress tests demonstrated that transgenic plants exhibited enhanced salt and freezing stress resistance compared with WT. Overexpressing the MYB-related gene *LlMYB3* in *Arabidopsis* transgenic plants enhanced tolerance to cold, drought, and salt stresses (Yong et al., 2019). The overexpression of MYB-related protein RSM1 of *Arabidopsis* and AmMYB1 of mangrove tree enhances the tolerance of seedlings to high salinity (Ganesan et al., 2012; Yang et al., 2018). A sheepgrass MYB-related transcription factor LcMYB2 can enhance the drought tolerance of transgenic plants (Zhao et al., 2019). Similar with the above reports, the peanut MYB-related protein AhMYB30 may play important role in freezing and salt stress regulation in *Arabidopsis*.

Transcription factors affect environmental stress tolerance of plants through the regulation of multiple downstream genes, and similarly MYB controls plant adaptation to environmental stresses through the regulation of genes downstream in the stress signaling pathways (Ambawat et al., 2013; Wang et al., 2021). Our data suggest that the overexpression of AhMYB30 protein results in enhanced transduction of stress-response signals. *ICE*, *CBF*, and *COR* genes, model an imperative ABA-independent signaling pathway, the *ICE*-*CBF*-*COR* cascade, a cold response pathway that alleviates cold stress in plants (Hwarari et al., 2022). Based on the quantitative fluorescent PCR results, the expression of *KIN1*, *RD29A* and *COR15A* in *AhMYB30*-overexpressing *Arabidopsis* were significantly higher than in WT plants. However, the expression of *ICE1* and three *CBFs* had no significant difference in the wild-type and transgenic plants. *KIN1*, *RD29A* and *COR15A* are known marker genes related to *ICE*-*CBF*-*COR* pathway, which have critical effects on regulating *Arabidopsis* responses to abiotic stresses (Hwarari et al., 2022). Different mechanisms are related to MYB TFs’ effect on modulating abiotic stress responses of plants. Certain cold-related genes, including *AtCBF1*, *AtCBF2*, *AtCBF3*, and *AtGSTU5*, are up-regulated within plants with *ZmMYB31* overexpression compared with WT plants (Li et al., 2019). The expression levels of *AtICE1*, *AtCOR47A*, *AtCBF3*, *AtCOR15B*, and *AtRD29A* were higher in the *RmMYB108* overexpression lines than in the WT (Dong et al., 2021). Besides, *GmMYB76* transgenic lines showed increased *RD29B*, *DREB2A*, *P5CS*, *RD1*, *ERD10*, and *COR78/RD29A* expression, and *GmMYB92* transgenic lines displayed increased *DREB2A*, *RD17*, and *P5CS* levels, whereas decreased *RD29B*, *COR6.6*, *COR15a* and *COR78/rd29A* levels (Liao et al., 2008). Moreover, MYB-related *LcMYB2* expression showed positive relation to some stress-responsive genes *LcLEA*, *LcDREB2c*, *LcMYB39*, and *Peroxidase 56* in drought-stressed sheepgrass (Zhao et al., 2019). The MYB-related LlMYB3 is possibly associated with anthocyanin biosynthetic pathway for regulating stress resistance of tiger lily (Yong et al., 2019). Our results indicated that AhMYB30 may be located downstream of CBFs and enhance the plant abiotic stress resistance through direct or indirect regulation of *KIN1*, *RD29A* and *COR15A* expression. The results are not completely consistent with any of the above studies, suggesting that AhMYB30 has its own unique regulatory mechanism.

In addition, the expression of *AhMYB30* was induced by exogenous ABA in peanut roots (Chen et al., 2014). The expression of *RD29B*, *ABI2*, *DREB2A*, *RD17*, *P5CS*, *ERD10*, *COR6.6*, *ERD11* and *COR78* increased within the *GmMYB177* transgenic plants (Liao et al., 2008). Among which, *ABI2* is a gene involved in ABA signaling pathway and its expression could be induced by cold stress (Kreps et al., 2002). The expression level of *ABI2* was also increased in *AhMYB30* transgenic *Arabidopsis* under both normal and cold conditions. The result indicated that AhMYB30 may also regulate plant abiotic stress tolerance through ABA-dependent pathway.

As an important oilseed crop, peanut had attracted increasing attention on the study of abiotic stress regulation. Based on gene expression response, TF families, like NAC, ERF, WRKY and MYB, are demonstrated to be involved in peanut abiotic stress regulation (Jiang et al., 2020). *AhERF6*, *AhERF019*, *AhbHLH112*, and so on, were all demonstrated to be associated with abiotic stresses in plants (Wan et al., 2014; Zhang et al., 2016; Li et al., 2021). However, due to the complexity of the peanut genome and the difficulties of genetic transformation, there are limited studies on the molecular mechanisms of abiotic stress regulation in peanut. More work needs to be performed in order to clarify the signaling pathway involved in abiotic stress regulation in peanut. Our studies indicated that the MYB-related transcription factor AhMYB30 increased salt and freezing stress resistance in transgenic plants through up-regulating some downstream stress-related gene levels within DREB/CBF and ABA-signaling pathways. Moreover, this work laid a theoretical foundation for the mining and functional studies of stress-related genes, and provided new gene resource for resistance molecular breeding in peanut. Future work will investigate the functions and mechanisms of AhMYB30 in peanut abiotic stress regulation.

## Conclusion

Peanut *AhMYB30* encode a MYB-related transcription factor, whose expression was induced by cold and salt stress. AhMYB30 has the basic characteristics of a transcription factor. AhMYB30 could enhance salt and freezing tolerance in transgenic Arabidopsis. AhMYB30 may exercise its function by up-regulating the expression of some downstream stress-related genes involved in DREB/CBF and ABA-signaling pathways.

## Data availability statement

The original contributions presented in the study are included in the article Material. Further inquiries can be directed to the corresponding author.

## Author contributions

Conceived and designed the total experiments: NC, XC. Performed the experiment: NC, LP, MS. Analyzed data: ZY, JX, XJ, FW. Manuscript writeup: NC, XY, TW. Funding procurement: XC. All authors contributed to the article and approved the submitted version.
